# The time course of disuse muscle atrophy of the lower limb in health and disease

**DOI:** 10.1002/jcsm.13067

**Published:** 2022-09-14

**Authors:** Edward J. O. Hardy, Thomas B. Inns, Jacob Hatt, Brett Doleman, Joseph J. Bass, Philip J. Atherton, Jonathan N. Lund, Bethan E. Phillips

**Affiliations:** ^1^ Department of General Surgery Royal Derby Hospital Derby UK; ^2^ Centre Of Metabolism, Ageing and Physiology (COMAP), School of Medicine University of Nottingham, Royal Derby Hospital Centre Derby UK; ^3^ Department of Anaesthetics Royal Derby Hospital Derby UK; ^4^ MRC‐Versus Arthritis Centre for Musculoskeletal Ageing Research (CMAR) and NIHR Nottingham Biomedical Research Centre Nottingham UK

**Keywords:** Muscle, Atrophy, Intensive care, Disuse, Inactivity

## Abstract

Short, intermittent episodes of disuse muscle atrophy (DMA) may have negative impact on age related muscle loss. There is evidence of variability in rate of DMA between muscles and over the duration of immobilization. As yet, this is poorly characterized. This review aims to establish and compare the time‐course of DMA in immobilized human lower limb muscles in both healthy and critically ill individuals, exploring evidence for an acute phase of DMA and differential rates of atrophy between and muscle groups. MEDLINE, Embase, CINHAL and CENTRAL databases were searched from inception to April 2021 for any study of human lower limb immobilization reporting muscle volume, cross‐sectional area (CSA), architecture or lean leg mass over multiple post‐immobilization timepoints. Risk of bias was assessed using ROBINS‐I. Where possible meta‐analysis was performed using a DerSimonian and Laird random effects model with effect sizes reported as mean differences (MD) with 95% confidence intervals (95% CI) at various time‐points and a narrative review when meta‐analysis was not possible. Twenty‐nine studies were included, 12 in healthy volunteers (total *n* = 140), 18 in patients on an Intensive Therapy Unit (ITU) (total *n* = 516) and 3 in patients with ankle fracture (total *n* = 39). The majority of included studies are at moderate risk of bias. Rate of quadriceps atrophy over the first 14 days was significantly greater in the ITU patients (MD −1.01 95% CI −1.32, −0.69), than healthy cohorts (MD −0.12 95% CI −0.49, 0.24) (*P* < 0.001). Rates of atrophy appeared to vary between muscle groups (greatest in triceps surae (−11.2% day 28), followed by quadriceps (−9.2% day 28), then hamstrings (−6.5% day 28), then foot dorsiflexors (−3.2% day 28)). Rates of atrophy appear to decrease over time in healthy quadriceps (−6.5% day 14 vs. −9.1% day 28) and triceps surae (−7.8% day 14 vs. −11.2% day 28), and ITU quadriceps (−13.2% day 7 vs. −28.2% day 14). There appears to be variability in the rate of DMA between muscle groups, and more rapid atrophy during the earliest period of immobilization, indicating different mechanisms being dominant at different timepoints. Rates of atrophy are greater amongst critically unwell patients. Overall evidence is limited, and existing data has wide variability in the measures reported. Further work is required to fully characterize the time course of DMA in both health and disease.

## Introduction

Maintenance of an adequate skeletal muscle mass is essential for a healthy, long life.^1^ It facilitates independence, locomotion and activities of daily living whilst also playing a key role in glucose homeostasis^2^ and the body's resilience to physiological stress.^3^ Low skeletal muscle strength and mass, with or without low physical performance, is known as sarcopenia^4^ and has been shown to be associated with an increased risk of falls, fractures, and the need for long term care.^5^ It is also associated with the prevalence of long‐term health conditions, such as type 2 diabetes,^6^ and a worse age‐matched risk of mortality.^7^

It is clear that some muscle loss inevitably occurs as part of the innate ageing process, with an average annual loss of 1% of muscle mass and 3% of strength after the age of 70.^8^ However, not all older people become sarcopenic, and identification of the underlying factors which drive certain individuals below a critical threshold of muscle mass is therefore a topic of considerable interest.^9^

There is evidence of disuse muscle atrophy (DMA) after just a few days of immobilization,^10^ and it is now increasingly suggested that intermittent episodes of acute DMA may contribute significantly to the development of sarcopenia.^11^ Repeated short periods of immobility become increasingly common with advancing age due to, for example, ill health, hospitalization, surgical recovery. The loss of muscle function caused by these episodes may lead to a reduction in habitual activity, followed by further episodes of acute DMA and a vicious cycle of punctuated dramatic loss of muscle mass which accelerates the usual age‐related changes.^11^

The adverse consequences of acute DMA are not limited to older adults, but also prolong recovery and delay return to normal activity following musculoskeletal injury, illness and surgery in all age groups.^12^ Despite this, the majority of research into DMA has been performed in the context of space exploration^13^ and consequently many studies report outcomes at only one timepoint and commonly after many weeks or even months of immobilization/disuse.^14^, ^15^ These findings may have little relevance to the effects of shorter periods of immobilization associated with illness, hospitalization and surgery.^16^, ^17^

Overall, the time course of DMA over shorter periods of immobilization, and how this varies between muscles of the leg is not well characterized. However, what evidence is available from healthy volunteer studies suggests that there may be a differential rate of DMA over the course of immobilization. More rapid loss of muscle mass^18^, ^19^ and function^18^ is reported in studies of shorter periods of immobilization, with slower rates seen in prolonged immobilization.^15^ This suggests that DMA may slow towards an eventual plateau^19^ as immobilization continues,^15^ with the most rapid loss of muscle in the initial period of disuse. The rate and extent of DMA also appears to differ between muscles and muscle groups,^20^, ^21^ suggesting that some muscles are more atrophy susceptible (aS) whilst others are more atrophy resistant (aR).^22^ Given the catabolic impact of illness, infection and inflammation,^23^, ^24^, ^25^ these findings are likely to be exaggerated in immobile hospitalized patients, resulting in more rapid and severe DMA than that seen in healthy volunteer studies.

Collectively these findings have many implications. Differential rates of DMA over time suggests different cellular mechanisms and pathways may dominate at different periods, and investigation of mechanisms behind the response of aR and aS muscles to immobilization may yield important insights into the mechanisms through which DMA is controlled and potentially mitigated. From a clinical perspective, if rates of atrophy are greatest at the start of immobilization, early introduction of strategies and therapies to counter this is essential during hospital admissions and other periods of disuse.

Therefore, the primary aim of this systematic review and meta‐analysis is to characterize the time‐course of DMA in muscles of the human lower limb during immobilization in both healthy and critically ill individuals. Secondary aims include comparison of atrophy rates in healthy and critically ill individuals, comparison of atrophy rates between different muscles and muscle groups of the leg, and exploration of the evidence for non‐linear muscle loss, including an acute phase of DMA.

## Methods

### Study design

This systematic review was registered prospectively with PROSPERO (registration number 106495) and carried out in accordance with the PRISMA statement.^26^ Any study reporting data on human lower limb muscle changes during immobilization or admission to ITU over multiple post‐immobilization timepoints was included. The minimum outcome reporting required for inclusion was measurement of at least one of (i) muscle volume; (ii) cross‐sectional area (CSA); (iii) lean leg mass; or (iv) muscle architecture (muscle thickness, fibre length, pennation angle) at baseline AND at a minimum of two timepoints following immobilization or admission to ITU. Studies which did not report on the above listed measures for leg muscle at multiple timepoints after immobilization or did not involve full immobilization, bed‐rest, or critical care admission, were excluded.

### Literature search

Literature searches were completed by a trained Clinical Research Librarian using the following databases: MEDLINE, Embase, CINHAL and CENTRAL (all searched from their inception to 01/04/2021). No language, publication type or date restrictions were applied to the searches. Previous systematic reviews of related topics were also searched for relevant studies. References of identified and potentially relevant studies were hand‐searched for further relevant studies. Finally, all studies citing the included studies identified on Google Scholar were screened for inclusion. Example search strategies can be found in Appendix S1.

Abstracts were screened independently by two authors (EH and TI) with the aid of Rayyan systematic review software (2016, Qatar Computing Research Institute, Doha, Qatar)^27^ and were considered for full‐text review if either author deemed them to be potentially relevant. A grey literature search as described above was also completed. Full‐text versions of all potentially relevant primary studies were then independently screened against the inclusion and exclusion criteria by two authors (EH and TI) and agreement for inclusion reached by consensus.

### Data extraction

Study characteristics and outcome data were extracted by one author (EH). Where studies reported the outcomes of interest in graphical form only, relevant data was extracted using WebPlotDigitiser.^28^ For studies reporting data as percentage change only, attempts were made to contact the authors to get original data. Risk of bias for included studies was assessed using the Risk of Bias In Non‐randomized Studies of Interventions (ROBINS‐I) assessment tool.^29^

### Statistical analysis

Effect sizes are reported as mean differences (MD) with 95% confidence intervals (CI). Change standard deviations (SD) were calculated by using the baseline SD and final SD and assuming a correlation coefficient of 0.7 using formulae from the Cochrane Handbook. Missing SD values were estimated from other studies. A DerSimonian and Laird random‐effects model was used. Statistical heterogeneity was assessed using the *I* ^2^ statistic. We attempted to conduct tests for publication bias, but the small number of studies precluded this. When studies from different cohorts measured the same outcome at similar timepoints, we performed a subgroup analysis and report the *P* value from the subgroup differences (*P* < 0.05). All meta‐analyses were conducted using Stata Version 16.

Where there was insufficient original data to allow formal comparison, or comparison was not possible due to mathematical constraints (e.g. between % change), pooled means were calculated from original data.

## Results

**Figure 1:**
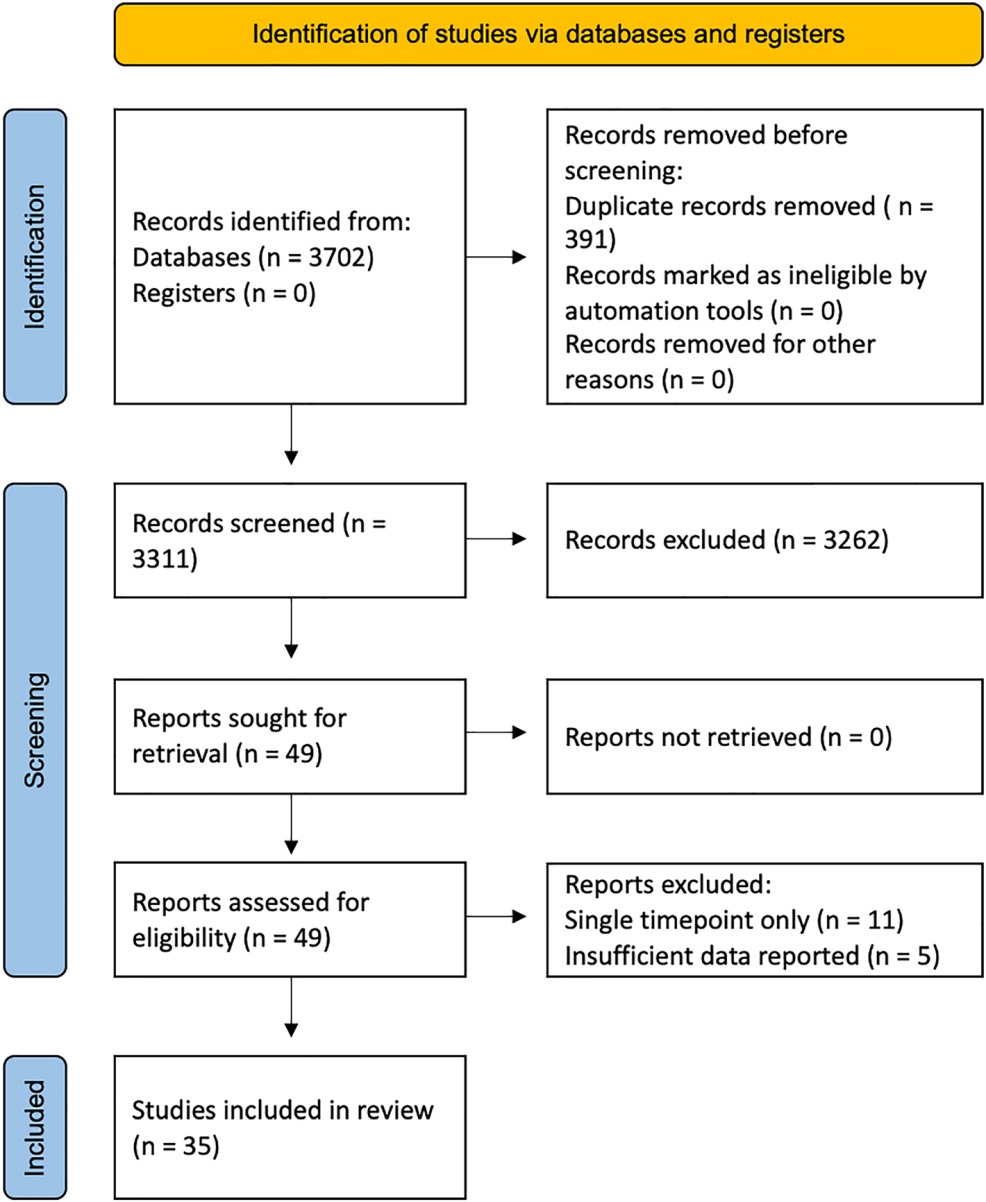
PRISMA flow diagram.^27^

A total of 3702 potentially relevant abstracted were screened for inclusion, of which 3311 were unique. Of the unique abstracts screened, 49 were found to be possibly relevant and underwent full‐text review. Following full‐text review, a further 14 were excluded, with the remaining 35 studies found to be relevant for inclusion in this review (Figure 1).

### Study characteristics

Characteristics of the included studies are shown in Table 1. The date of publication ranged from 1997 to 2020. Thirty‐two studies were full‐texts, one was a correspondence and two were conference abstracts; all were published in peer‐reviewed journals.

**Table 1:** Summary of included studies

Study	Type of study/publication	Setting	Mechanism of immobilization	N	Muscles studied	Imaging modality	Measurements made	Measurement timepoints (day)
Abe T (1997)	Cohort. Full paper	Healthy	Bed Rest	8	Ant thigh (grouped), Calf (grouped), VL, MG	USS	Muscle Thickness (mm)	7, 14, 20
Akima (1997)	Cohort. Full paper	Healthy	Bed Rest	10	Knee Extensors (grouped), Knee Flexors (Grouped), VL VM, VI, RF, MG, LG, Sol, TA	MRI	Vol (cm^3^), CSA (cm^2^)*	10, 20
Annettan (2017)	Cohort. Full paper	ITU (trauma)	Critical care	38	TA, RF	USS	CSA (cm^2^), MT (cm)	5, 10, 15, 20
Armbrecht (2010)	RCT. Effect of resistive vibration exercise (RVE). Full paper	Healthy	Bed Rest	20	Whole leg	DXA	Lean Mass (g)	12, 31, 44, 55
Belavy (2009)	RCT. Effect of RVE. Full paper	Healthy	Bed Rest	20	TA, MG, LG, Sol, Peroneals, Tib Post, FDL, Quads, Hamstring, RF, Vastii, Adductors	MRI	Vol (% change)	14, 28, 42, 56
Boer (2007)	Cohort. Full paper	Healthy	ULLS	17	Quads (grouped), VM, VL, VI, RF	MRI, USS	CSA, FL, PA	14, 23
Borges (2019)	Cohort. Full paper	ITU (sepsis)	Critical care	37	RF	USS	CSA (cm^2^)	2, 4, 6, 10
Cartwright (2012)	Cohort. Full paper	ITU (raspatory failure)	Critical care	16	TA, RF	USS	MT (cm)	3, 7, 10
Dilani (2009)	Cohort. Full paper	Healthy	Bed Rest	12	Ant hip muscles, IL, PS, ILP, RF	MRI	CSA (cm^2^)	14, 28, 42, 56
Hayes (2018)	Cohort. Full paper	ITU (ECMO)	Critical care	25	RF, VL, VI, RF, Quads (grouped)	USS	CSA (cm^2^), MT (cm), Echogenicity	10, 20,
Hirose (20013)	Case control vs Electrical muscle stimulation (EMS). Full paper	ITU (CVA/TBI)	Critical care	11	Ant Thigh (grouped), Post Thigh (grouped), Ant lower leg (grouped), Post Lower leg (grouped)	CT	CSA (cm^2^)	7, 21, 28, 35,42
Kawashima (2004)	Cohort. Full paper	Healthy	Bed Rest	10	Adductors (grouped), AM, AL, AB	MRI	CSA (cm^2^)	10, 20,
Kilroe (2020)	Cohort. Full paper	Healthy	ULLS	13	Quads (grouped)	MRI	Vol (cm^3^)	2, 7
Katari (2018)	Cohort. Full paper	ITU (all causes)	Critical care	100	RF	USS	MT (cm)	3, 5, 7, 10, 14, 21
Menids (2009)	RCT, vs. RVE. Full paper	Healthy	Bed Rest	6	Iliacus, Psoas, Iliopsoas, Sartorius, RF	MRI	CSA (cm^2^)	14, 28, 42, 56
Mikovic (2012)	Cohort. Full paper	Healthy	Bed Rest	9	AB, AL, AM, Pec, Gracilis, Sart, RF, Vasti (grouped), MH, LH, EDL, TA, Perioneals, FDL, FHL, TP, LG, MG, Sol	MRI	Vol (cm^3^)	28, 56
Mulder (2006)	RCT, vs RVE. Full paper	Healthy	Bed Rest	10	Quad (grouped)	MRI	CSA (cm^2^)*	Low
Nakanishi (2017)	Cohort. Full paper	ITU (all causes)	Critical care	28	RF	USS	CSA (% change), MT (% change)	3, 5, 7
Pardo (2018)	Cohort. Full paper	ITU (mixed)	Critical care	29	Quads (grouped)	USS	MT (cm)	3, 5, 7, 10, 21
Parry (2015)	Cohort. Full paper	ITU (mixed)	Critical care	22	RF, VL, VI	USS	CSA, MT, PA (all % change)	3, 5, 7, 10
Psatha (2012)	Cohort. Full paper	Ankle Fracture	ULLS (below knee cast)	18	TA, Sol, GM, GL, Tricep Surae (grouped)	MRI	CSA (%change)*	8, 15, 29, 43
Putuchery (2017)	Cohort. Correspondence	ITU (all causes)	Critical care	43	RF	USS	CSA, MT (all % change)*	7, 10
Rittweger (2005)	Cohort. Full paper	Healthy	Bed Rest	9	Calf Muscles (combined)	CT	CSA (% change)*	28, 43, 56, 68, 88
Segaran (2017)	Cohort. Full paper	ITU	Critical care	44	Bicep, Forearm, Thigh (average of all)	US	MT (cm)	7, 14
Seynnes (2008)	Cohort. Full paper	Healthy	ULLS	8	Sol, MG, LG	MRI, USS	Vol (cm^3^), FL, PA	14, 28
Silva (2019)	Cohort. Full paper	ITU (TBI)	Critical care	30	TA, RF	USS	MT (cm)*	3, 7, 14
Stevens (2004)	Cohort. Full paper	Ankle fracture	ULLS (below knee cast)	20	LG, MG, Sol, Ant lower leg (combined), Post lower leg (combined)	MRI	CSA (cm^2^)	7, 14
Shin (2016)	Cohort. Full paper	ITU (all causes)	Critical care	27	Upper arm, Thigh, Calf	Tape	Limb circumference	3, 5
ten Haaf (2017)	Cohort. Full paper	ITU (mixed)	Critical care	14	Quads (combined)	USS	CSA (% change)	7, 14, 21, 28
Toledo (2017)	Cohort. Abstract only	ITU (all causes)	Critical care	20	Quads (combined)	USS	MT (cm)	3, 7
Turton (2016)	Cohort. Full paper	ITU (all causes)	Critical care	22	MG, VL	USS	MT, FL, PA	5, 10
Twose (2018)	Cohort. Full paper	ITU (mixed)	Critical care	26	RF	USS	CSA (cm^2^)	3, 5, 7, 10
Vendenborne (1998)	Cohort. Full paper	Ankle Fracture	ULLS (below knee cast)	1	MG, LG, Sol	MRI	CSA (cm^2^)	14, 28
Wapel (2017)	RCT vs EMS. Abstract only	ITU (all causes)	Critical care	15	Thigh muscle (combined)	CT	CSA (%change)	7, 14

RCT, randomized controlled trial; RVE, resistive vibration exercise; EMS, electrical muscle stimulation; ITU, intensive treatment unit; ULLS, unilateral lower limb suspension; Quads, quadriceps; VL/M/I, vastus lateralis; medialis; intermedius (respectively); RF, rectus femoris; M/LG, medial/lateral gastrocnemius; Sol, soleus; TA, tibialis anterior; Vol, volume; CSA, cross‐sectional area; MT, muscle thickness.

* Data only presented in graphical format and extracted using web plot digitizer.

Twelve studies reported results from healthy volunteer studies. Of these, three used unilateral lower limb suspension (ULLS) as a means of immobilization, whilst the other nine involved full bed‐rest. Eight were cohort studies, and the remaining four consisted of data taken from the control limb of a randomized control trial (RCT). Sample sizes ranged from 6 to 20. Time to first post‐immobilization measurement ranged from 2 to 28 days, and time to final measurement ranged from 7 to 88 days.

Eighteen studies reported results from patients admitted to an ITU. Sixteen were cohort studies, with data from the control limb of one case control study and one RCT. Twelve studies contained a mixture of all ITU admissions, two were of patients admitted with traumatic brain injury (TBI), one study was in patients with sepsis, one in patients with respiratory failure, and one in patients having extracorporeal membrane oxygenation (ECMO). Sample sizes ranged from 11 to 100. Time to first post‐immobilization measurement ranged from 2 to 10 days, and time to final measurement ranged from 5 to 42 days.

Three studies reported results from patients immobilized with a below knee cast following ankle fracture. All were cohort studies. Sample size ranged from 1 to 20. Time to first post‐immobilization measurement ranged from 7 to 14 days, and time to final measurement ranged from 14 to 43 days.

### Risk of bias

Risk of bias was assessed using the ROBINS‐I tool (Table 2). Overall, 33 studies were found to be at moderate risk of bias, and 2 studies were found to be at low risk of bias. Of those studies found to be at moderate risk of bias, all were at moderate risk in measurement of outcomes due to a lack of blinding in assessors performing or analysing the scans. Three studies performed in ITU patients were at moderate risk of bias due to patient selection, because of varying time from start of intervention (ITU admission) to baseline scans. Nine studies were at serious risk of bias due to missing data. Eight of these studies were in ITU patients, with a loss of patients as time progressed, and one was in patients following ankle fracture with not all patients attending for scans at all timepoints. One healthy volunteer study was at moderate risk of bias due to deviation from intended intervention, as immobilized patients performed tests of maximum voluntary contraction at 2 timepoints during their immobilization.

**Table 2:** ROBINS I risk of bias assessment for included studies

Study	Confounding	Selection	Classification of intervention	Deviation from intended intervention	Missing data	Measurement of outcomes	Reported result	Overall
Abe T (1997)	Low	Low	Low	Low	Low	Moderate	Low	Moderate
Akima (1997)	Low	Low	Low	Low	Low	Moderate	Low	Moderate
Annettan (2017)	Low	Low	Low	Low	Low	Moderate	Low	Low
Armbrecht (2010)	Low	Low	Low	Low	Low	Moderate	Low	Moderate
Belavy (2009)	Low	Low	Low	Low	Low	Low	Low	Low
Boer (2007)	Low	Low	Low	Moderate	Low	Moderate	Low	Moderate
Borges (2019)	Low	Low	Low	Low	Low	Moderate	Low	Moderate
Cartwright (2012)	Low	Low	Low	Low	Serious	Moderate	Low	Moderate
Dilani (2009)	Low	Low	Low	Low	Low	Moderate	Low	Moderate
Dillon (2018)	Low	Low	Low	Low	Low	Moderate	Low	Moderate
Hayes (2018)	Low	Moderate	Low	Low	Low	Moderate	Low	Moderate
Hirose (2013)	Low	Low	Low	Low	Low	Moderate	Low	Moderate
Kawashima (2004)	Low	Low	Low	Low	Low	Moderate	Low	Moderate
Kilroe (2020)	Low	Low	Low	Low	Low	Moderate	Low	Moderate
Kitari (2018)	Low	Low	Low	Low	Low	Moderate	Low	Moderate
Menids (2009)	Low	Low	Low	Low	Low	Moderate	Low	Moderate
Mikovic (2012)	Low	Low	Low	Low	Low	Low	Low	Low
Mulder (2006)	Low	Low	Low	Low	Low	Moderate	Low	Moderate
Nakanishi (2017)	Low	Low	Low	Low	Serious	Moderate	Low	Moderate
Pardo (2018)	Low	Low	Low	Low	Low	Moderate	Low	Moderate
Parry (2015)	Low	Moderate	Low	Low	Serious	Moderate	Low	Moderate
Psatha (2012)	Low	Low	Low	Low	Serious	Moderate	Low	Moderate
Putuchery (2013)	Low	Low	Low	Low	Low	Moderate	Low	Moderate
Rittweger (2005)	Low	Low	Low	Low	Low	Moderate	Low	Moderate
Segaran (2017)	Low	Moderate	Low	Low	Serious	Moderate	Low	Moderate
Seynnes (2008)	Low	Low	Low	Low	Low	Moderate	Low	Moderate
Silva (2018)	Low	Low	Low	Low	Serious	Moderate	Low	Moderate
Silva (2019)	Low	Low	Low	Low	Serious	Moderate	Low	Moderate
Stevens (2004)	Low	Low	Low	Low	Low	Moderate	Low	Moderate
ten Haaf (2017)	Low	Low	Low	Low	Low	Moderate	Low	Low
Toledo (2017)	Low	Low	Low	Low	Low	Moderate	Low	Moderate
Turton (2016)	Low	Low	Low	Low	Serious	Moderate	Low	Moderate
Twose (2018)	Low	Low	Low	Low	Serious	Moderate	Low	Moderate
Vendenborne (1998)	Low	Low	Low	Low	Low	Moderate	Low	Moderate
Wapel (2017)	Low	Low	Low	Low	Low	Moderate	Low	Moderate

### Healthy volunteer studies

#### Whole leg

One study^30^ reporting whole‐leg changes as assessed by DXA in 20 immobilized healthy participants showed that lean mass changed by −3.5% at day 12 (D12), −5.4% at D31, −7.0% at D44 and −8.3% by D56.

#### Hip flexors

One study,^31^ in which six healthy young males had 56 days of bed‐rest, reported the results of immobilization on the CSA of the iliacus and psoas as assessed by MRI. CSA remained stable across all timepoints, with no documented loss seen in either the psoas or iliacus. Compared with baseline measurements iliopsoas CSA decreased by −2.78% at D14, −4.86% at D28 and −2.78% at D42.

#### Quadriceps

Eight studies^20^, ^31^, ^32^, ^33^, ^34^, ^35^, ^36^, ^37^ reported changes in quadriceps muscles during immobilization. Six studies (total 63 participants) used bed rest and two studies (total 30 participants) used unilateral limb suspension (ULS). One study^36^ reported changes in anterior thigh muscle thickness, four reported changes in quadriceps CSA^31^, ^32^, ^37^, ^38^ and four reported changes in quadriceps volume.^20^, ^32^, ^33^, ^34^ Combined quadriceps muscle volume decreased by −1.7% at D3, −5.0% at D7, −5.71% to −6.5% by D10 to D14, −7.31% at D20, −9.1% by D28, − 12% by D42 and −14.4% by D56. Rates of atrophy in individual quadricep muscles varied, with smaller losses observed in rectus femoris (−3.5% to −4.1% at D14 and −5.1% to −7.4% at D56) compared with vastus muscles (−4.7% to −6.7% at D14 and −5.6 to −15.9% at D56).

**Figure 2:**
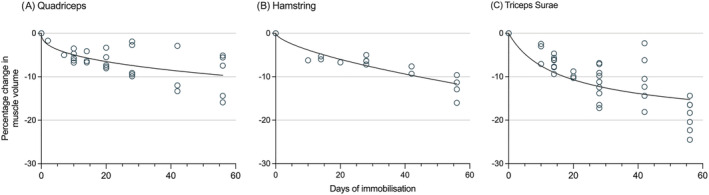
Graphical representation of the median % change in muscle volume during immobilization with line of best fit. (A) Quadriceps, (B) hamstring, and (C) triceps Surae; each as reported in the healthy volunteer studies included in this review.

Combined quadriceps CSA changes corelated with volume changes. There was a decrease of −3.9 to −5.9% at D10–14, −7.6% to −10.0% at D20, −7.6% at D28, −10.3% at D42 and −13.6% by D56. A full breakdown of quadriceps volume and CSA changes are available in Tables S1 and S2. Figure 2A illustrates the time‐course of changes in quadriceps muscle volume based on the studies included this review.^31^, ^32^, ^37^, ^38^ Anterior thigh muscle thickness, reported by only one study, decreased by −7.1% by D7, −12.6% by D14 and −12.0% by D20.

#### Hamstrings

Four studies^20^, ^32^, ^33^, ^34^ reported the changes observed in knee flexor muscles during immobilization. Three studies (total 39 participants) used bed‐rest and one study (total 13 participants) used ULS. Two studies reported changes in hamstring CSA,^32^, ^34^ whilst all four reported changes in muscle volume.^20^, ^32^, ^33^, ^34^ Combined hamstring muscle volume decreased by −1.4% at D3, −2.1% at D7, −6.0% to −6.21% by D10 to D14, −6.69% at D20, −6.3% to −7.19% by D28, −9.3% by D42 and −11.3% to −16.0% by D56. Full details of hamstring volume changes are shown in Table S3. Hamstring CSA decreased by −1.7% to −2.38% at D3, −4.0% to −5.9% at D7, −9.3% at D10 and −9.3% at D20. Figure 2B illustrates the time‐course of changes in hamstring muscle volume based on the studies included this review.^20^, ^32^, ^33^, ^34^

#### Plantar flexors

Six studies^20^, ^32^, ^33^, ^36^, ^39^, ^40^ reported the changes observed in plantar flexor muscles during immobilization. Five studies (total 55 participants) used bed rest and one study (total 8 participants) used ULS. One study^36^ reported changes in combined calf muscle thickness, and observed decreases of −7.1% at D7, −8.2% at D14 and −6.9% at D_2_O. Four studies reported changes in plantar flexor volume.^20^, ^32^, ^33^, ^40^ Combined triceps surae muscle volume decreased by −7.8% at D14, −11.2% at D28, −14.4% at D42 and −18.3% by D56. Rates of atrophy in individual triceps surae muscles varied. In general, smaller losses were observed in the lateral belly of the gastrocnemius (−2.4% to −7.7% at D14 and −14.4% to −16.5% at D56) compared with the medial gastrocnemius and soleus which showed similar rates of atrophy (−3.0% to −9.4% at D14 and −20.4 to −22.3% at D56). Full results of plantar flexor volume changes are shown in Table S4. Figure 2C illustrates the time‐course of changes in triceps surae muscle volume based on the studies included this review.^20^, ^32^, ^33^, ^40^

#### Dorsiflexors

Three studies^20^, ^32^, ^33^ (total 39 participants) reported changes in dorsiflexor muscles during bed‐rest immobilization. All three studies reported changes in tibialis anterior volume, with wide variation in results between studies. Decreases in volume ranged from −0.7% to −9.2% at D14 and 0.8% to −7.7% at D28.

### Ankle fracture patients

**Figure 3:**
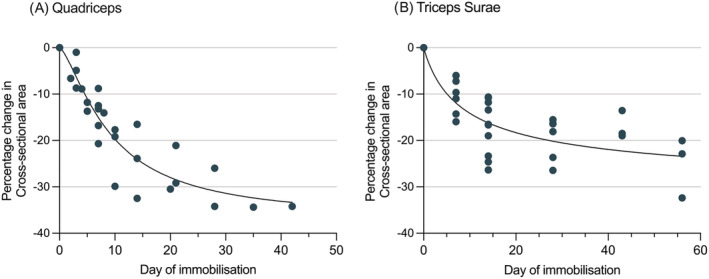
Graphical representation of the median % change in muscle CSA during immobilization with line of best fit. (A) Quadriceps muscles of intensive treatment unit (ITU) patients, and (B) triceps Surae muscles of patients following ankle fracture; both as reported in the studies included in this review.

Three studies^41^, ^42^, ^43^ with a total of 39 patients reported the changes in lower leg musculature during immobilization in plaster cast following ankle fracture. All three studies reported change in CSA of triceps surae muscles, with a decrease of −6.0% to −16.0% by D7, −10.6% to −26.4% at D14, −15.5% to −26.5% by D28, −13.5% to −18.9% by D43 and −32.4% by D56. A full breakdown of changes in plantar flexor CSA in ankle fracture patients is available in Table S5. Figure 3B illustrates the time‐course of changes in plantar flexor CSA in ankle fracture patients based on the studies included this review.^41^, ^42^, ^43^ One study^41^ reported changes in tibialis anterior CSA, with a loss of −3.08% by D8, −6.2% at D15, −10.5% at D28, and −10.3% by D43.

### ITU patients

Eighteen studies reported changes in lower limb muscle size during ITU admission. Thirteen studies reported changes in quadriceps (eight measured CSA^44^, ^45^, ^46^, ^47^, ^48^, ^49^, ^50^ and eight measured muscle thickness^44^, ^46^, ^51^, ^52^, ^53^, ^54^, ^55^), two reported changes in plantar flexors (1 CSA,^47^ 1 muscle thickness^53^), four reported changes in dorsiflexors (1 CSA,^47^ 1 muscle thickness^56^) and three reported change in hamstring CSA.^47^ Three studies measured combined quadriceps CSA^47^, ^50^ and observed a decrease of −13.2% by D7 and −23.9% to −32.5% on D14. The remaining five studies measured rectus femoris CSA and reported changes of −1.0% to −8.7% at D3, −8.8 to −13.7% at D5, −12.5% to −20.7% by D7, and −17.7% to −29.9% by D14. Figure 3A illustrates the time‐course of changes in quadriceps muscle CSA in ITU patients based on the studies included this review.^44^, ^45^, ^46^, ^47^, ^48^, ^49^, ^50^ A full breakdown of the results from papers included in this study which report changes in muscles of ITU patients is given in Tables S6–S8.

### Comparative analysis

#### Healthy versus ITU

##### Meta‐analysis

Meta‐analysis for change in rectus femoris CSA after 14 days of immobilization revealed a mean difference (MD) of −0.12 (95% CI: −0.49 to 0.24) in healthy participants (*I* ^2^ = 0%) (Figure S1), whereas in ITU patients the corresponding MD was −1.01 (95% CI: −1.32 to −0.69) (*I* ^2^ = 84%) (Figure S2). On sub‐group analysis this indicates a statistically significant greater loss of muscle in ITU patients (*P* < 0.001), although it should be noted that changes in ITU patients were subject to considerable heterogeneity.

#### Analysis of raw data

**Figure 4:**
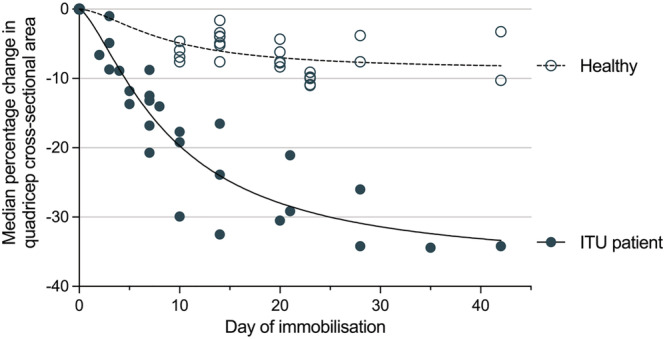
Graphical representation of the median % change in quadriceps CSA during immobilization with line of best fit. Intensive treatment unit (ITU) patients shown as closed circles and healthy volunteers shown as open circles); both as reported in the studies included in this review.

To allow an illustrative comparison, pooled means were calculated for quadricep muscle CSA at each timepoint for ITU patients and healthy immobilized volunteers. These results show that ITU patients experienced dramatically more muscle loss than healthy immobilized individuals, with a − 4.6% loss of CSA at D2 (vs. −1.6%), −13.9% loss at D7 (vs. −5.6%), and −18.7% at D10–14 (vs. −5.5%). Figure 4 illustrates this difference.

#### Healthy versus ankle fracture

**Figure 5:**
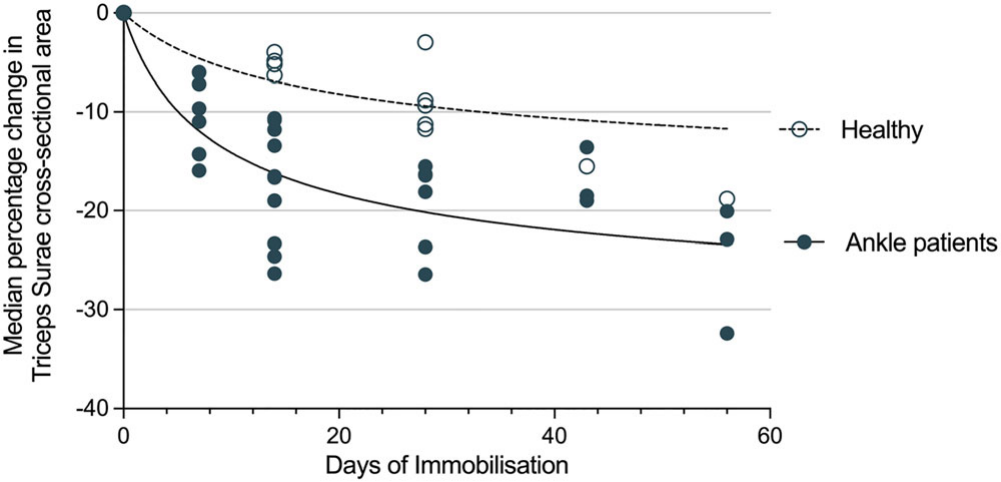
Graphical representation of the median % change in triceps Surae CSA during immobilization with line of best fit. Ankle fracture patients shown as closed circles and healthy volunteers shown as open circles; both as reported in the studies included in this review.

There was insufficient original data to allow formal meta‐analysis of the difference between muscle loss in immobilized healthy individuals and patients immobilized following ankle fracture. However, the data suggests that muscle loss tends to be greater in patients following ankle fracture than in healthy individuals. Triceps surae muscle volume decreased by −2.4% to −9.4% by D14 and −6.8% to −17.2% by D28 in healthy participants, whereas muscle CSA decreased by −10.6% to −26.4% by D14 and −15.5% to −26.5% by D28 in patients following ankle fracture. Comparison of these changes are illustrated in Figure 5.

#### Comparison between muscle groups

**Figure 6:**
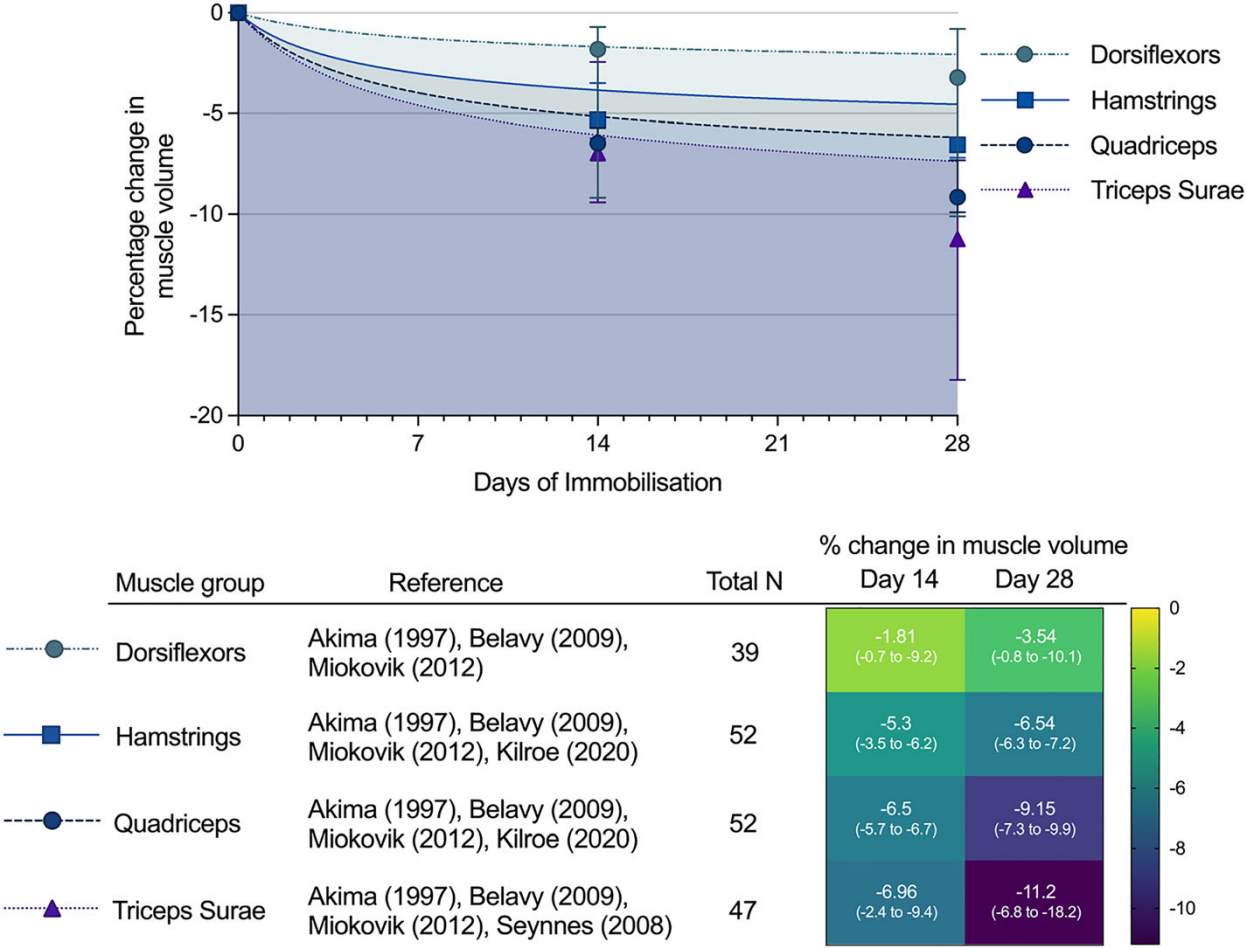
Illustrative comparison of muscle atrophy rates in different lower limb muscle groups of immobilized healthy volunteers. Based on pooled means calculated from studies included in this review. Range of changes displayed in parentheses.

Formal comparison of the rate of muscle atrophy between muscle groups was not possible as many studies reported % change only, with insufficient original data to allow meta‐analysis. Pooled means of the change in muscle volume of each muscle group in healthy participants were therefore calculated to allow illustrative comparison. These results show that tibialis anterior had the lowest rates of atrophy with −1.8% loss at D14 and −3.2% loss at D28, followed by the hamstring muscles (−5.3% at D14, −6.5% D28) and quadriceps (−6.5% D14, −9.2% D29). Triceps surae showed the greatest losses with −6.96% loss D14 and −11.2% loss at D28. Figure 6 illustrates the difference in rates of muscle atrophy between muscle groups of the lower limb, with full results in Table S9.

Similar trends were seen in patients following ankle fracture. In the one paper reporting two muscles in this patient group^41^ TA CSA reduced by −3.1% by D8 and −6.2% by D15, whereas triceps surae reduced by −6.0% to −7.2% at D8 and −10.8% to −16.7% at D15.

### Time course of disuse muscle atrophy

There was insufficient original data to allow formal meta‐analysis of the difference in rates of atrophy over the time‐course of immobilization. Analysis of pooled means revealed that when averaged over D0 to D14, quadriceps muscle volume of healthy volunteers decreased by an average of −0.46%/day, whereas when averaged from D0 to D28 the rate decreases to −0.33%/day. Similarly, for the triceps surae of healthy volunteers, the rate of atrophy from D0 to D14 is −0.50%/day whereas for D0 to D28 the rate is −0.40%. In the quadriceps of ITU patients the rate of atrophy when averaged over D0 to D2 is −2.3%/day, from D0 to D7 is −1.99%, and from D0 to D14–1.34%. These figures suggest that rates of muscle atrophy decrease over time, with more accelerated atrophy in critically unwell patients.

### Measurement method

Formal comparison of the rate of muscle atrophy as defined by different measurements (muscle thickness vs. CSA vs. volume) through meta‐analysis was not possible due to mathematical constraints, as many studies reported % change only without original measurements, resulting in insufficient comparable original data. Percentage change of the pooled means of original data were therefore calculated to allow an illustrative comparison. In healthy volunteers' quadriceps muscle volume decreased by −5.6% by D10–14 and −7.4% by D20–28, whereas CSA decreased by −5.5% and −7.9% respectively. In ITU patient's quadriceps CSA decreased by −18.7% by D10–14, whereas muscle thickness decreased by −20.4%.

## Discussion

Despite the large amount of research into skeletal muscle atrophy we have found there is limited evidence to characterize the true time‐course of DMA in human lower limb muscles in both healthy volunteers and critically unwell patients, with few studies reporting multiple post‐immobilization timepoints. The 12 healthy volunteer studies reported combined results from 140 participants, and the 18 ITU studies a total of 516 patients. Studies varied widely in the muscle groups studied, the measurements used, and the timepoints reported, resulting in limited comparable data. Most studies were at moderate risk of bias, most commonly due to lack of blinding of the assessors. Several ITU studies were also at risk of bias due to high dropout rates as time progressed.

### Rates of muscle atrophy

Immobilized healthy muscles start to atrophy quickly, with one study reporting a significant decrease in quadriceps volume after just 2 days.^34^ Rates of muscle atrophy are significantly greater in critically unwell patients, with changes in rectus femoris CSA being more than 2.5 times greater in ITU patients than in healthy participants. Whilst this is to be expected due to the presence of potent catabolic drivers such as severe systemic inflammation^57^ and starvation^58^ in ITU patients, rates of atrophy in patients immobilized following ankle fracture also appear to be greater than in healthy volunteers. Whilst patients following ankle fracture are not subject to the same severe systemic inflammation as ITU patients, localized inflammatory responses may occur, which may contribute to the increased rates of DMA. A further possible explanation is the degree of immobilization that muscles undergo. Following ankle fracture (or ULS for healthy volunteers), legs are totally immobilized in a cast preventing even minimal, non‐weight bearing contractile activity which is possible in bed‐rest studies.

Rates of atrophy appear to slow as duration of immobilization progresses. This finding is consistent across all muscle groups and is observed in healthy volunteers, ITU patients and following ankle fracture. This finding is in keeping with observations from other studies,^19^ where rates of atrophy follow a pattern of exponential decay with the greatest losses in the first 14 days, slowing over time to reach an eventual nadir.^59^ This consistent finding suggests that the mechanisms involved in disuse atrophy may vary over the duration of immobilization and that they are at their most potent in the acute early phase (i.e., day 0 to day 14). It appears that muscle mass maintenance is dependent on continued contractile activity, is lost rapidly upon cessation of this, and rates of atrophy slow once muscle mass is near its intrinsic set point, unless another catabolic factor is present.

### Differential atrophy rates between muscles

In ITU patients results from muscle groups other than the quadriceps are limited and no definite variability in rates of atrophy between muscle groups can be identified. However, in healthy volunteers' triceps surae and quadriceps muscle groups appear to be the most susceptible to disuse, atrophying at around 3‐times the rate of ankle dorsiflexors, such as the tibialis anterior, which appear to be the most atrophy resistant. This is in keeping with observations made in other studies,^21^, ^22^, ^60^ and may reflect a trend towards greater atrophy in those muscle which usually contribute the most force during standing and walking.^59^

There is also some evidence of differential rates of atrophy of individual muscles within a muscle group, although it should be noted that this finding is based on a limited number of studies, with not all studies showing consistent findings. For example, in muscle of healthy volunteers, the vastii of the quadriceps muscle group appears to be more atrophy susceptible than the rectus femoris.^20^, ^32^, ^33^ These findings mirror those of other studies not included in this review^61^, ^62^ which report variable rates of muscle atrophy within muscle groups following amputation and tendon rupture. If proven, the notion of differential rates of atrophy in individual muscles within a muscle group raises some questions for the proposition of immobility or inactivity being the main driver behind age‐related muscle loss, as the proportion of each individual muscle within the quadriceps seems to be maintained with advancing age.^63^

Formal comparison of changes in different muscle measurements in the assessment of atrophy was outside the scope of this review. However, from the results included in this review, changes in muscle volume and CSA in the quadriceps of healthy volunteers, and CSA and muscle thickness in the rectus femoris of ITU patients, appear to give broadly similar results. This contrasts with the findings of other studies which have suggested that muscle thickness underestimates atrophy in ITU patients when compared with CSA.^64^, ^65^, ^66^

As multiple studies have demonstrated that acute DMA is accompanied by a corresponding, but even greater loss of muscle function,^18^ the findings in this review have important clinical implications, especially as in most patients DMA will be accelerated by inflammation and poor nutrition. Further research is required into the mechanisms at work during the acute phase of DMA and potentially strategies to counteract it. Beyond optimizing patient care to allow and encourage early mobilization, techniques such as bed‐based resistance and vibration exercise, and electrical muscle stimulation have shown some promise in the reduction of DMA.^67^, ^68^

### Limitations

The major limitation of the current analysis concerns the analysis of raw data from the included studies. Unlike formal meta‐analysis, this data does not take account of the fact that individuals within each study are more likely to be similar than those in other studies (patients with sepsis in one study combined with TBI patients) and therefore needs to be interpreted with caution.

Furthermore, despite ultrasound measurements being validated in multiple studies various factors may impact of the quality of data acquisition.^69^, ^70^, ^71^ Whilst it is beyond the scope of this review to analyse the imaging data acquisition techniques of each individual study, we recognize that results based on data obtained without use a gold standard imaging technique must be interpreted with caution.

Finally, as this review intended to characterize the time‐course of disuse muscle atrophy in muscles of the human lower limb during immobilization in both healthy and critically ill individuals, we chose to only include studies with measures of muscle mass at multiple timepoints (beyond baseline) to best reflect the true temporality of DMA (versus studies which only report baseline and 1 other timepoint). We acknowledge that this approach will have excluded a number of studies reporting muscle mass losses with disuse; however, alternative recent reviews with a differing search strategy do synthesize the evidence from these (e.g. previous study^72^).

## Conclusion

In conclusion, further work is required to fully characterize the time course of DMA in the human lower limb in both health and disease. However, results from the studies included in this review suggest that DMA occurs rapidly, with the highest rate of muscle loss in the most acute phase, and that these changes are significantly greater in the critically unwell patient. Both findings highlight the importance of early intervention to minimize muscle loss, especially in unwell patients. Further, rates of DMA appear to vary both between muscle groups and between individual muscles within a muscle group, an observation that must be considered during intervention design.

## Ethical statement

The authors certify that they comply with the ethical guidelines for authorship and publishing in the *Journal of Cachexia, Sarcopenia and Muscle*.^73^

## Funding

The research was funded by the Medical Research Council and Versus Arthritis via the MRC‐Versus Arthritis Centre for Musculoskeletal Ageing Research.

## Conflict of interest

Edward Hardy, Thomas Inns, Jacob Hatt, Brett Doleman, Joseph Bass, Philip Atherton, Jonathan Lund and Bethan Phillips declare they have no conflicts of interest.

## Supporting information


**Appendix S1.** Supporting Information

**Table S1:** Percentage change in Quadriceps muscle volume in immobilised young healthy volunteers.
Table S2: Percentage change in Quadriceps muscle cross‐sectional area (CSA) in immobilised young healthy volunteers.
Table S3: Percentage change in Hamstring muscle volume in immobilised young healthy volunteers.
Table S4: Percentage change in Triceps Surae muscle volume in immobilised young healthy volunteers.
Table S5: Percentage change in Triceps Surae muscle cross‐sectional area (CSA) in patients immobilised following ankle fracture.
Table S6: Summary of % change in Quadriceps muscle thickness (MT) in intensive treatment unit (ITU) patients.
Table S7: Summary of % change in Quadriceps cross‐sectional area (CSA) in intensive treatment unit (ITU) patients.
Table S8: Summary of changes in other leg muscles of intensive treatment unit (ITU) patients.
Table S9: Pooled mean change of muscle volume for different muscle groups in healthy volunteers after 14 and 28 days of immobilisation. Range of changes displayed in parentheses.
Figure S1: Forrest plot of change in Quadriceps cross‐sectional area (CSA) in healthy volunteers between baseline and day 14.
Figure S2: Forrest plot of change in Quadriceps cross‐sectional area (CSA) in intensive treatment unit (ITU) patients between baseline and day 14.
