# Supplementary material for: The time course of disuse muscle atrophy of the lower limb in health and disease
Source: J Cachexia Sarcopenia Muscle. 2022 Sep 14;13(6):2616–29. doi: 10.1002/jcsm.13067 (PMC9745468; doi:10.1002/jcsm.13067)
Supplement: Supplementary file 1 — Appendix S1. Supporting Information [file JCSM-13-2616-s002.pdf]

Medline Search Strategy

#	Database	Search term	Results
1	Medline	LEG/	61949
2	Medline	exp "HAMSTRING MUSCLES"/	351
3	Medline	exp "MUSCLE, SKELETAL"/	255196
4	Medline	exp MUSCLES/	646327
5	Medline	*ATROPHY/ OR *"MUSCULAR ATROPHY"/	9757
6	Medline	exp ATROPHY/ OR exp "MUSCULAR ATROPHY"/	42154
7	Medline	exp TIME/ OR exp "TIME FACTORS"/	1349631
8	Medline	(2 OR 3 OR 4)	646327
9	Medline	(1 AND 8)	9954
10	Medline	(5 OR 6)	42154
11	Medline	(9 AND 10)	248
12	Medline	(7 AND 11)	21
13	Medline	exp IMMOBILIZATION/	25990
14	Medline	exp "MUSCULAR ATROPHY"/	12810
15	Medline	exp *"MUSCULAR ATROPHY"/	8595
16	Medline	(14 OR 15)	12810
17	Medline	(1 AND 13 AND 16)	17
18	Medline	(7 AND 17)	1
19	Medline	11 [Human age groups Adult	175

		OR Aged] [Humans]	
20	Medline	12 [Human age groups Adult OR Aged] [Humans]	17
21	Medline	17 [Human age groups Adult OR Aged] [Humans]	9
22	Medline	18 [Human age groups Adult OR Aged] [Humans]	1
23	Medline	((((leg OR thigh OR calf OR lower limb) AND muscle) AND atrophy) AND (immobil* OR inactiv* OR reduced activity OR bed rest)).ti,ab	162
24	Medline	((((leg OR thigh OR calf OR lower limb) AND muscle) AND atrophy) AND (immobil* OR inactiv* OR reduced activity OR bed rest)).ti,ab,af	251
25	Medline	exp "LOWER EXTREMITY"/ OR exp LEG/ OR exp THIGH/	155966
26	Medline	(13 AND 14 AND 25)	35
27	Medline	23 [Human age groups Adult OR Aged] [Humans]	77
28	Medline	24 [Human age groups Adult OR Aged] [Humans]	136
29	Medline	26 [Human age groups Adult OR Aged] [Humans]	19
30	Medline	exp "BED REST"/	3801
31	Medline	(1 AND 14 AND 30)	16
32	Medline	(7 AND 31)	4
33	Medline	31 [Human age groups Adult OR Aged] [Humans]	12

34	Medline	32 [Human age groups Adult OR Aged] [Humans]	3
