# Supplementary material for: The time course of disuse muscle atrophy of the lower limb in health and disease
Source: J Cachexia Sarcopenia Muscle. 2022 Sep 14;13(6):2616–29. doi: 10.1002/jcsm.13067 (PMC9745468; doi:10.1002/jcsm.13067)
Supplement: Supplementary file 2 — Table S1: Percentage change in Quadriceps muscle volume in immobilised young healthy volunteers. Table S2: Percentage change in Quadriceps muscle cross‐sectional area (CSA) in immobilised young healthy volunteers. Table S3: Percentage change in Hamstring muscle volume in immobilised young healthy volunteers. Table S4: Percentage change in Triceps Surae muscle volume in immobilised young healthy volunteers. Table S5: Percentage change in Triceps Surae muscle cross‐sectional area (CSA) in patients immobilised following ankle fracture. Table S6: Summary of % change in Quadriceps muscle thickness (MT) in intensive treatment unit (ITU) patients. Table S7: Summary of % change in Quadriceps cross‐sectional area (CSA) in intensive treatment unit (ITU) patients. Table S8: Summary of changes in other leg muscles of intensive treatment unit (ITU) patients. Table S9: Pooled mean change of muscle volume for different muscle groups in healthy volunteers after 14 and 28 days of immobilisation. Range of changes displayed in parentheses. Figure S1: Forrest plot of change in Quadriceps cross‐sectional area (CSA) in healthy volunteers between baseline and day 14. Figure S2: Forrest plot of change in Quadriceps cross‐sectional area (CSA) in intensive treatment unit (ITU) patients between baseline and day 14. [file JCSM-13-2616-s001.docx]

## Supplementary Data

| **Author** | **N** | **Muscle** | **% Change in Quadriceps muscle volume** | | | | | | |
| --- | --- | --- | --- | --- | --- | --- | --- | --- | --- |
|  |  |  | **D2** | **D7** | **D10-14** | **D20** | **D28** | **D42** | **D56** |
| **Akima (1997)** | 10 | Quadriceps  (combined) | - | - | -5.71 | -7.31 | - | - | - |
| **Belavy (2009)** | 20 | Quadriceps  (combined) | - | - | -6.5  (^+^/_-_3.5) | - | -9.1  (^+^/_-_3.4) | -12.0  (^+^/_-_3.4) | -14.4  (^+^/_-_3.5) |
| **Kilroe (2020)** | 13 | Quadriceps  (combined) | -1.7  (^+^/_-_0.3) | -5.0  (^+^/_-_0.6) | - | - | - | - | - |
|  | | | | | | | | | |
| **Akima (1997)** | 10 | Vastus  (combined) | - | - | -4.7 to  -6.7 | -5.5 to  -8.0 | - | - | - |
| **Belavy (2009)** | 20 | Vastus  (combined) |  |  | -6.7  (^+^/_-_3.7) | - | -9.9  (^+^/_-_3.6) | -13.3  (^+^/_-_3.5) | -15.9  (^+^/_-_3.7) |
| **Miokovic (2012)** | 9 | Vastus  (combined) | - | - | - | - | -9.4 | - | -5.6 |
|  | | | | | | | | | |
| **Akima (1997)** | 10 | Rectus Femoris | - | - | -3.5 | -3.3 | - | - | - |
| **Belavy (2009)** | 20 | Rectus Femoris | - | - | -4.1%  (^+^/_-_3.5) | - | -2.7  (^+^/_-_3.5) | -2.9  (^+^/_-_3.4) | -5.1  (^+^/_-_3.5) |
| **Miokovic (2012)** | 9 | Rectus Femoris | - | - | - | - | -1.8 | - | -7.4 |

**Supplementary Table 1: Percentage change in Quadriceps muscle volume in immobilised young healthy volunteers.**

| **Author** | **N** | **Muscle** | **% Change in Quadriceps CSA** | | | | |
| --- | --- | --- | --- | --- | --- | --- | --- |
|  |  |  | **D10-14** | **D20-23** | **D28** | **D42** | **D56** |
| **Akima (1997)** | 10 | Quadriceps (combined) | -5.9 | -7.6 | - | - | - |
| **Mulder (2008)** | 10 | Quadriceps (combined) | -3.9 | - | -7.6 | -10.2 | -13.6 |
| **de Boer (2007)** | 17 | Quadriceps (combined) | -5.2 | -10.0 | - | - |  |
|  | | | | | | | |
| **Akima (1997)** | 10 | Vastus (combined) | -4.6 to -7.6 | -6.2 to -8.3 | - | - | - |
| **de Boer (2007)** | 17 | Vastus (combined) | -3.4 to -7.6 | -9.1 to -11.0 | - | - |  |
|  | | | | | | | |
| **Akima (1997)** | 10 | Rectus Femoris | -4.6 | -4.3 | - | - | - |
| **Menids (2009)** | 6 | Rectus Femoris | -1.6 | - | -3.8 | -3.2 | -5.4 |
| **de Boer (2007)** | 17 | Rectus Femoris | -4.0 | -10.9 | - | - | - |

**Supplementary Table 2: Percentage change in Quadriceps muscle cross-sectional area (CSA) in immobilised young healthy volunteers.**

| **Author** | **N** | **Muscle** |  | **% Change in Hamstring muscle volume** | | | | |
| --- | --- | --- | --- | --- | --- | --- | --- | --- |
|  |  |  | **D7** | **D10-14** | **D20** | **D28** | **D42** | **D56** |
| **Akima (1997)** | 10 | Hamstrings (combined) | - | -6.2 | -6.7 | - | - | - |
| **Belavy (2009)** | 20 | Hamstrings  (combined) | - | -6.0  (^+^/_-_3.3) | - | -6.4  (^+^/_-_3.2) | -9.3  (^+^/_-_3.2) | -11.3  (^+^/_-_3.1) |
| **Kilroe (2020)** | 13 | Hamstrings (combined) | -3.5 | - | - | - | - | - |
| **Miokovic (2012)** | 9 | Medial Hamstrings | - | - | - | -7.2 | - | -16.0 |
| **Miokovic (2012)** | 9 | Lateral Hamstrings | - | - | - | -6.3 | - | -12.9 |

**Supplementary Table 3: Percentage change in Hamstring muscle volume in immobilised young healthy volunteers.**

| **Author** | **N** | **Muscle** | **% Change in Triceps Surae muscle volume** | | | | |
| --- | --- | --- | --- | --- | --- | --- | --- |
|  |  |  | **D10-14** | **D20** | **D28** | **D42** | **D56** |
| **Belavy (2009)** | 20 | Triceps Surae (combined) | -7.8  (^+^/_-_1.8) | - | -11.2  (^+^/_-_1.8) | -14.4  (^+^/_-_1.8) | -18.3  (^+^/_-_2.0) |
|  |  |  |  |  |  |  |  |
| **Akima**  **(1997)** | 10 | Medial Gastrocnemius | -3.00 | -9.93 | - | - | - |
| **Belavy (2009)** | 20 | Medial Gastrocnemius | -9.4  (^+^/_-_1.5) | - | -13.8  (^+^/_-_1.6) | -18.1  (^+^/_-_1.1) | -22.3  (^+^/_-_1.5) |
| **Miokovic (2012)** | 9 | Medial Gastrocnemius | - | - | -16.50 | - | -20.41 |
| **Seynnes (2008)** | 8 | Medial Gastrocnemius | -6.09 | - | -9.79 | - | - |
|  |  |  |  |  |  |  |  |
| **Akima**  **(1997)** | 10 | Lateral Gastrocnemius | -2.43 | -10.28 | - | - | - |
| **Belavy (2009)** | 20 | Lateral Gastrocnemius | -7.7  (^+^/_-_3.8) | - | -11.2  (^+^/_-_2.9) | -10.5  (^+^/_-_1.8) | -14.4  (^+^/_-_2.8) |
| **Miokovic (2012)** | 9 | Lateral Gastrocnemius | - | - | -12.44 | - | -16.46 |
| **Seynnes (2008)** | 8 | Lateral Gastrocnemius | -5.69 | - | -7.09 | - | - |
|  |  |  |  |  |  |  |  |
| **Akima**  **(1997)** | 10 | Soleus | -7.05 | -8.76 | - | - | - |
| **Belavy (2009)** | 20 | Soleus | -6.2  (^+^/_-_1.8) | - | -9.1  (^+^/_-_1.8) | -12.3  (^+^/_-_1.8) | -16.5  (^+^/_-_1.8) |
| **Miokovic (2012)** | 9 | Soleus | - | - | -17.22 | - | -24.51 |
| **Seynnes (2008)** | 8 | Soleus | -4.66 | - | -6.84 | - | - |

**Supplementary Table 4: Percentage change in Triceps Surae muscle volume in immobilised young healthy volunteers.**

| **Author** | **N** | **Muscle** | **% Change in Triceps Surae muscle CSA** | | | | |
| --- | --- | --- | --- | --- | --- | --- | --- |
|  |  |  | **D7** | **D14** | **D28** | **D42** | **D56** |
| **Stevens (2004)** | 20 | Posterior lower leg (combined) | -9.66 | -18.98 | - | - | - |
|  |  |  |  |  |  |  |  |
| **Psatha**  **(2012)** | 18 | Medial Gastrocnemius | -7.2 | -16.7 | -23.6 | -18.5 | - |
| **Stevens (2004)** | 20 | Medial Gastrocnemius | -16.0 | -26.4 | - | - | - |
| **Vandenborne**  **(1998)** | 1 | Medial Gastrocnemius | - | -13.4 | -18.1 | - | -22.89 |
|  |  |  |  |  |  |  |  |
| **Psatha**  **(2012)** | 18 | Lateral Gastrocnemius | -6.0 | -11.8 | -16.31 | -13.6 | - |
| **Stevens (2004)** | 20 | Lateral Gastrocnemius | -11.0 | -24.6 | - | - | - |
| **Vandenborne**  **(1998)** | 1 | Lateral Gastrocnemius | - | -16.5 | -26.5 | - | -32.4 |
|  |  |  |  |  |  |  |  |
| **Psatha**  **(2012)** | 18 | Soleus | -6.0 | -10.8 | -16.4 | -19.0 | - |
| **Stevens (2004)** | 20 | Soleus | -14.3 | -23.3 | - | - | - |
| **Vandenborne**  **(1998)** | 1 | Soleus | - | -10.6 | -15.5 | - | -20.1 |

**Supplementary Table 5: Percentage change in Triceps Surae muscle cross-sectional area (CSA) in patients immobilised following ankle fracture.**

| **Author** | **N** | **Muscle** |  |  |  |  |  |
| --- | --- | --- | --- | --- | --- | --- | --- |
|  |  |  | **% Change in Quadriceps MT** | | | | |
|  |  |  | **D3** | **D5** | **D7** | **D10-14** | **D20** |
| **Segaran (2017)** | 17 | Quadriceps (combined) | 0 to -2.7 | 0 to -5.0 | -6.7 to -16.0 | - | - |
| **Toledo (2017)** | 20 | Quadriceps (combined) | -2.59 | - | -12.07 | - | - |
| **Pardo (2018)** | 29 | Quadriceps (combined) | -0.49 | -5.1 | -14.8 | -20.9 | -22.7 |
| **Hayes (2018)** | 25 | Quadriceps (combined) | - | - | - | -9.0 | -30.3 |
|  | | | | | | | |
| **Annettan (2017)** | 40 | Rectus Femoris | - | -4.65 | - | -6.98 | -11.63 |
| **Cartwright (2012)** | 16 | Rectus Femoris | -7.95 | - | 17.42 | -1.89 | - |
| **Parry (2015)** | 22 | Rectus Femoris | -8.7 | -16.6 | -24.9 | -30.4 | - |
| **Katari (2018)** | 100 | Rectus Femoris | -8.0 | - | -11.0 | - | - |
| **Hayes (2018)** | 25 | Rectus Femoris | - | - | - | -25.4 | -34.9 |
|  | | | | | | | |
| **Parry (2015)** | 22 | Vastus Lateralis | -0.2 | -5.7 | -6.0 | -14.1 | - |
| **Turton (2016)** | 22 | Vastus Lateralis | - | -8.5 | - | -22.9 | - |
| **Hayes (2018)** | 25 | Vastus Lateralis | - | - | - | -16.4 | -32.5 |
| **Parry (2015)** | 22 | Vastus Intermedialis | -1.3 | -18.1 | -20.0 | -29.7 | - |
| **Hayes (2018)** | 25 | Vastus Intermedialis | - | - | - | -16.4 | -31 |

**Supplementary Table 6: Summary of % change in Quadriceps muscle thickness (MT) in intensive treatment unit (ITU) patients.**

| **Author** | **N** | **Muscle** |  | **% Change in Quadriceps muscle CSA** | | | | | | | |
| --- | --- | --- | --- | --- | --- | --- | --- | --- | --- | --- | --- |
|  |  |  | **D3** | **D5** | **D7** | **D10-14** | **D15** | **D21** | **D28** | **D35** | **D42** |
| **Hirose (2013)** | 1 | Quadriceps  (combined) | - | - | -13.2 | -23.9 | - | -29.1 | -34.2 | -34.4 | -34.2 |
| **Ten Haaf (2017)** | 14 | Quadriceps  (combined) | - | - | -8.77 | -16.53 | - | -21.09 | -26 | - | - |
| **Wapel (2018)** | 15 | Quadriceps  (combined) | - | - | -13.2 | -32.5 | - | - | - | - | - |
|  |  |  |  |  |  |  |  |  |  |  |  |
| **Annettan**  **(2017)** | 38 | Rectus Femoris | - | -3.28 | - | -16.39 | -24.59 | -42.62 | - | - | - |
| **Nakanishi (2017)** | 28 | Rectus Femoris | -8.70 | -13.7 | -20.7 | - | - | - | - | - | - |
| **Parry (2015)** | 22 | Rectus Femoris | -1.0 | -11.8 | -16.8 | -29.9 | - | - | - | - | - |
| **Putuchery (2013)** | 42 | Rectus Femoris | -4.9 | - | -12.5 | -17.7 | - | - | - | - | - |
| **Twose (2018)** | 26 | Rectus Femoris | -4.0 | -7.0 | -10.5 | -14.9 | - | - | - | - | - |
| **Borges (2019)** | 37 | Rectus Femoris | -6.61 | -8.88 | -14.04 | - | - | - | - | - | - |
| **Hayes**  **(2018)** | 25 | Rectus Femoris | - | - | - | -19.2 | - | -30.5 | - | - | - |

**Supplementary Table 7: Summary of % change in Quadriceps cross-sectional area (CSA) in intensive treatment unit (ITU) patients.**

| **Paper** | **N** | **Muscle** | **Measure** |  | **% Change** | | | | | | | |
| --- | --- | --- | --- | --- | --- | --- | --- | --- | --- | --- | --- | --- |
|  |  |  |  | D3 | D5 | D7 | D10 -14 | D15 | D21 | D28 | D35 | D42 |
| **Annettan**  **(2017)** | 38 | Tibialis Anterior | CSA | - | -3.28 | - | -16.39 | -24.59 | -42.62 | - | - | - |
| **Annettan**  **(2017)** | 38 | Tibialis Anterior | MT | - | -4.65 | - | -6.98 | -6.98 | -11.63 | - | - | - |
| **Cartwright**  **(2012)** | 16 | Tibialis Anterior | MT | 5.14 | - | 8.88 | 12.15 | - | - | - | - | - |
| **Hirose (2015)** | 1 | Posterior lower leg | CSA | - | - | -6.85 | -17.11 | - | -22.37 | -26.13 | -28.01 | -28.76 |
| **Turton (2016)** | 22 | Gastrocnemius | MT | - | -3.88 | - | -11.63 | - | - | - | - | - |
| **Hirose**  **(2015)** | 1 | Anterior lower leg | CSA | -2.42 | - | -15.41 | -26.13 | - | -34.74 | -39.47 | -37.97 | -30.76 |
| **Silva (2018)** | 22 | Tibialis Anterior | MT | - | - | -10.05 | -20.11 | - | - | - | - | - |
| **Silva (2018)** | 30 | Tibialis Anterior | MT | - | - | -8.67 | -13.93 | - | - | - | - | - |
| **Hirose**  **(2012)** | 1 | Posterior thigh | CSA | - | - | -12.0 | -22.7 | - | -28.4 | -32.0 | -33.8 | -35.9 |

**Supplementary Table 8: Summary of changes in other leg muscles of intensive treatment unit (ITU) patients.**


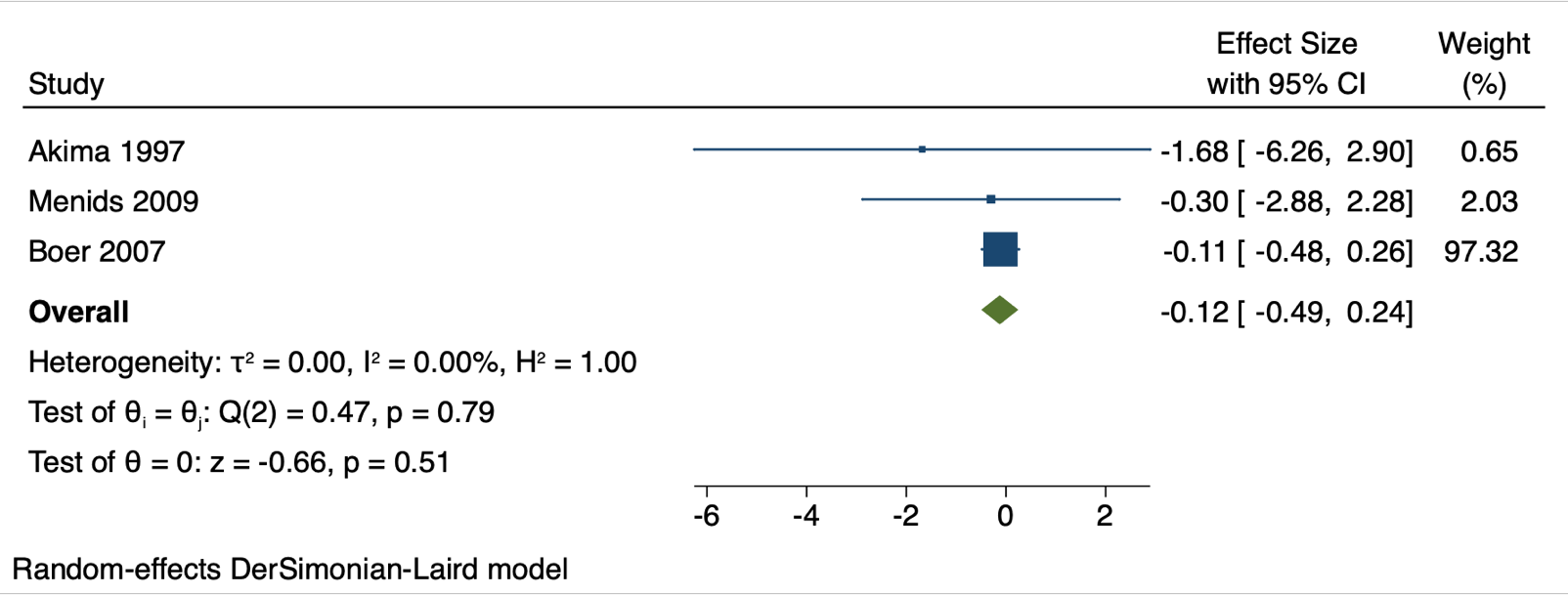


**Supplementary Figure 1: Forrest plot of change in Quadriceps cross-sectional area (CSA) in healthy volunteers between baseline and day 14.**


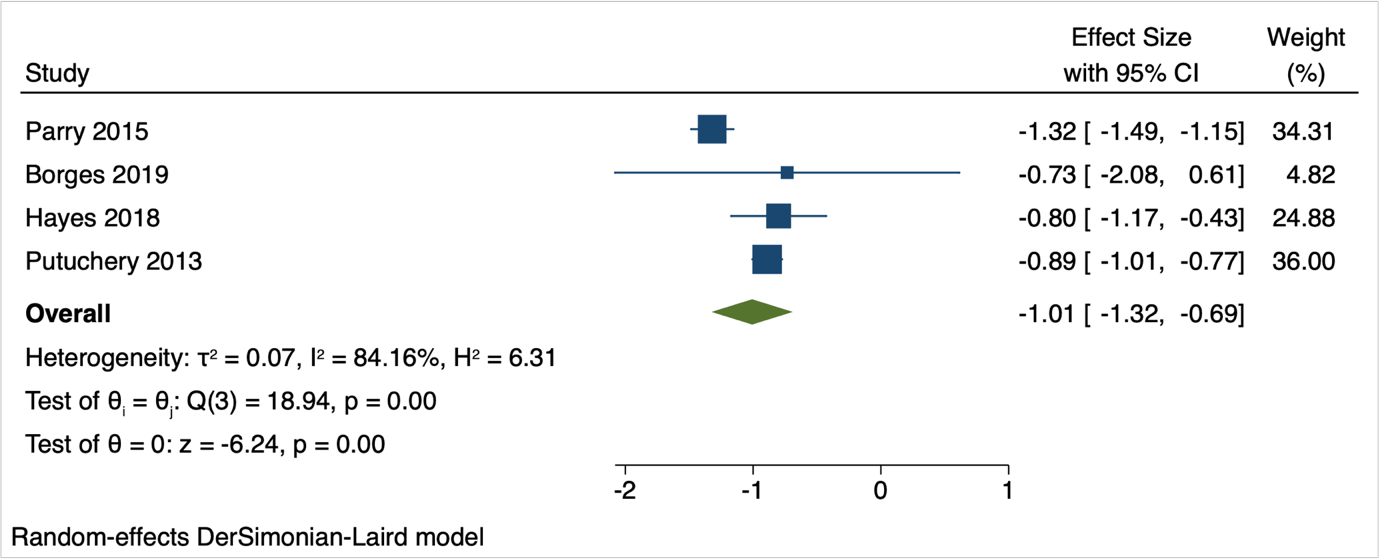


**Supplementary Figure 2: Forrest plot of change in Quadriceps cross-sectional area (CSA) in intensive treatment unit (ITU) patients between baseline and day 14.**

| **Muscle Group** | **Papers** | **Total N** | **% change in muscle volume** | |
| --- | --- | --- | --- | --- |
|  |  |  | **D14** | **D28** |
| Quadriceps | Akima (1997), Belavy (2009), Miokovik (2012), Kilroe (2020) | 52 | -6.5  (-5.7 to -6.7) | -9.15  (-7.3 to –9.9) |
| Hamstrings | Akima (1997), Belavy (2009), Miokovik (2012), Kilroe (2020) | 52 | -5.3  (-3.5 to -6.2) | -6.54  (-6.3 to -7.2) |
| Triceps Surae | Akima (1997), Belavy (2009), Miokovik (2012), Seynnes (2008) | 47 | -6.96  (-2.4 to -9.4) | -11.2  (-6.8 to -18.2) |
| Dorsiflexors | Akima (1997), Belavy (2009), Miokovik (2012) | 39 | -1.81  (-0.7 to -9.2) | -3.2  (-0.8 to -10.1) |

**Supplementary Table 9: Pooled mean change of muscle volume for different muscle groups in healthy volunteers after 14 and 28 days of immobilisation.** Range of changes displayed in parentheses.
